# Explicit Mechanistic Causal Analyses or Interventional Trials Are Required for Objective, Clinical, Voice-Based Parkinson Disease Characterization

**DOI:** 10.2196/111711

**Published:** 2026-09-28

**Authors:** Max Little

**Affiliations:** 1 School of Computer Science, University of Birmingham, Edgbaston, Birmingham, England, B15 2TT, United Kingdom, 44 121 414 3344

**Keywords:** digital voice analysis, causal inference, diagnostic trials, machine learning, confounding

## Abstract

For most with Parkinson disease, a movement disorder, voice and speech are impaired at some point, which presents an opportunity to use digital recordings and sophisticated machine learning to assist objective clinical characterization of the condition. However, as highlighted by Shukla et al in their August 20, 2026 paper, ad-hoc observational datasets typically contain spurious causal associations that escape a purely statistical analysis. This commentary argues that causal inference, and ultimately, diagnostic clinical trials, are required to establish a direct mechanistic relationship between disease and algorithm predictions.

Shukla et al [1] demonstrate that naive statistical analysis of digital recordings to assist objective, clinical, voice-based characterization of Parkinson disease can be highly misleading, even with sophisticated machine learning algorithms. However, this is a consequence of ad hoc observational datasets, which typically contain spurious causal associations that escape a purely statistical analysis. Causal inference, and ultimately, diagnostic clinical trials, are required to establish a direct mechanistic relationship between disease and algorithm predictions.

There has been considerable interest, stretching back 30 or more years, in using voice and/or speech for objective clinical characterization of certain disorders and diseases that cause vocal signs and symptoms [2]. Sounds are readily captured noninvasively by ubiquitous digital recording equipment, and recent progress in machine learning (ML) algorithms has enabled nearly fully automated processing of these digital signals to predict clinical labels such as positive/negative diagnoses or symptom severity by training these algorithms on cohort datasets (Figure 1A). Movement disorders such as Parkinson disease are prime examples of clinical conditions that are inherently amenable to this approach.

**Figure 1.**
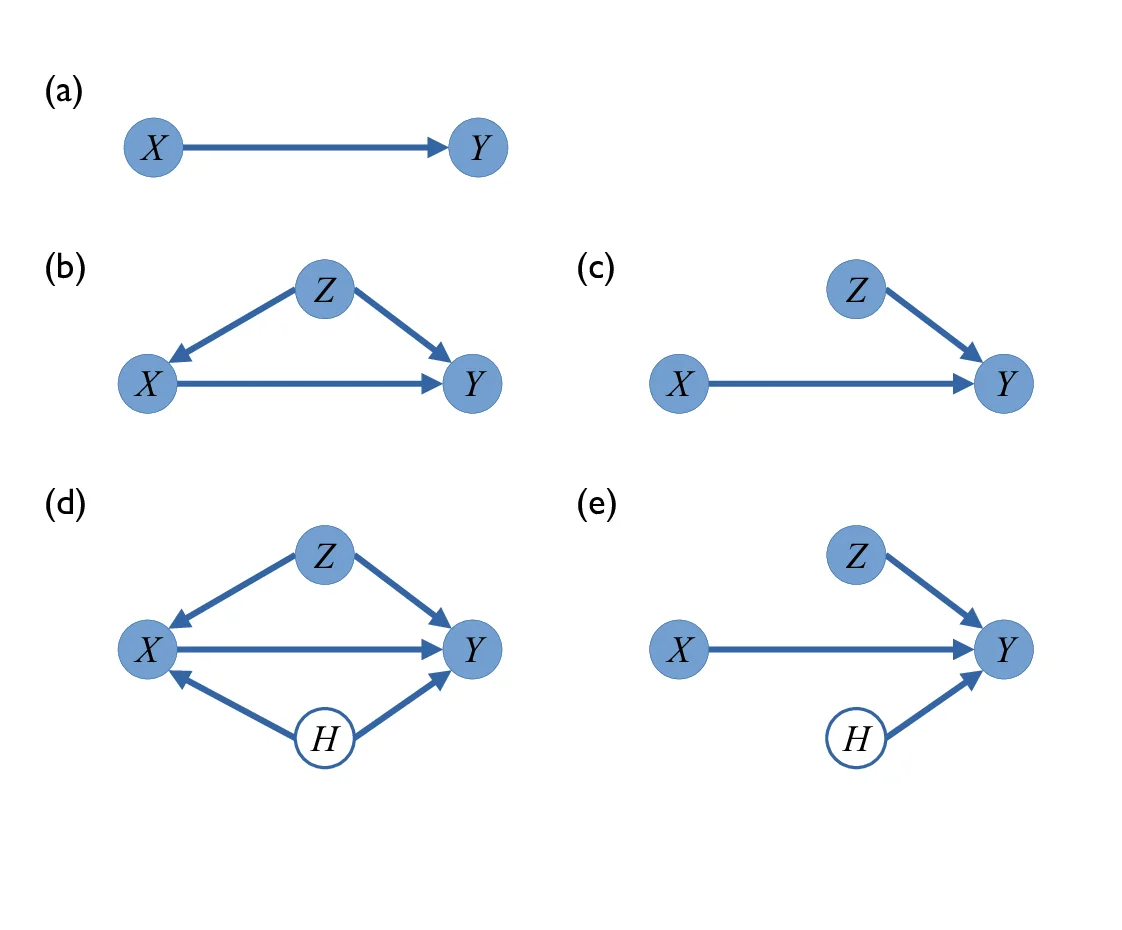
(A) Naive machine learning prediction of clinical disease status (X) from digital voice/speech features (Y) assumes features are solely dependent upon the underlying disease process and nothing else. (B) The situation is generally more structurally complex, however: age, recording environment, and/or identity are very often common causes of both the digital recordings and disease status, so they are causal confounders (Z). (C) Causal inference can break the spurious association path this entails. (D) Nevertheless, causal inference is not always possible, for instance, where there is hidden confounding (H). (E) In that situation, only an interventional trial can obtain a scientifically defensible estimate of machine learning prediction performance.

Nonetheless, a naive application of these algorithms overlooks common statistical issues with these observational datasets, which are typically collected ad hoc absent principled experimental design protocols. For instance, there may be imbalanced ages or differences in acoustic environments between cases and controls. These imbalances can be sufficiently large that a simple benchmark prediction based on age or environment can be more accurate than predictions based on the digital audio itself. Shukla et al [1] demonstrate exactly this phenomenon with a specific dataset (Bridge2AI-Voice), using a sophisticated deep learning transformer architecture applied to the digital audio features.

This is clearly scientifically dubious because objective characterization should be based on the audio recordings of the patients, not on irrelevances such as patient age or acoustic setting. Bayesian analysis can be used for rebalancing via a suitable choice of prior, but it is specific to the particular dataset [3]. Majority/minority resampling methods can compensate for the imbalance by modifying the data itself. These either repeat samples, introducing statistical artifacts, or discard data, which reduces the available data size and thereby increases the statistical uncertainty of estimation for these ML models.

However, the problems above, arising from ad hoc data collection settings, often run deeper. Age can affect voice and speech (presbyphonia), and many diseases and syndromes become more common with advancing age. Age can thus be a common cause of both clinical label and vocal features [4]. Similarly, individuals have distinct vocal identities, and many diseases of interest are chronic or progressive and do not undergo spontaneous remission, so datasets may have no recordings of individuals in both control and case state, even when they contain multiple recordings from the same individual [5]. Thus, particularly in small cohorts, both clinical label and vocal features have another common cause: the individual’s uniqueness. An algorithm must detect the *direct* causal relationship between vocal signs and symptoms and clinical label, but these nuisance causes produce *spurious* associations that easily swamp sensitive ML algorithms [5].

This mechanistic framing gives a rigorous structural causal meaning (Figure 1B) to confounding, loosely used by Shukla et al [1]. Causal problems such as this are, in general, provably beyond the reach of purely statistical analysis [4]; indeed, cross-validation (CV) [6] cannot correct for causal confounding [5]. Stratified (individual-level) CV [1,6] attempts to resolve issues such as identity, age, or sex confounding, but this generally biases the quantification of prediction error because, by construction, the training and test sets no longer have the same distribution, which invalidates hold-out methods like CV [3,5,6].

Solving causal confounding in these datasets can be achieved through explicit causal inference methods (Figure 1C), the simplest of which (applicable in the backdoor case) is controlling for confounders by including them as covariates in the prediction model [4]. Covariate controlling is unsatisfactory, however, because the algorithm then requires the value of the confounder at prediction time. Probabilistic adjustment methods remove this restriction, but this comes at the expense of requiring an explicitly probabilistic model of all joint variables in the problem [4]. Many sophisticated ML algorithms (such as deep learning) are not explicitly probabilistic, and modeling high-dimensional digital audio data is difficult. Shukla et al [1] use propensity score matching, applicable in the backdoor case, which constructs a subsample that approximately mimics the causal consequences of removing the association between confounders and clinical label (Figure 1C). However, discarding data like this always increases statistical uncertainty and demands positivity, which is violated in deterministic confounding such as the spurious identity/vocal uniqueness association and in small datasets in general [7,8]. If such adjustment is possible then counterfactual inference may be used to test more complex predictions beyond the scope of purely interventional causal logic, such as hypothetical case/control assignments for specific individuals [4].

Increasingly sophisticated causal machine learning is one route to more scientifically defensible ML modeling in this setting, but causal inference demands sufficiently precise knowledge of the structural causal mechanisms involved. There can be hidden factors (Figure 1D) such that only in certain causal arrangements is the direct effect identifiable from the observational data [4]. Thus, ultimately, explicitly interventional data collection (eg, through randomized controlled trials) is required to completely eliminate bias due to spurious associations (Figure 1E). However, the usual problem to solve in this setting is predicting clinical labels or scores, and it is impossible (or unethical) to impose this status on trial participants as an intervention. Thus, we must settle for diagnostic trials [9] where treatment outcome is generally used as the effect, and the intervention is the use of the novel voice/speech-based prediction algorithm versus the current standard-of-care.

Clinical trials like this are, in theory, the gold standard test of digital voice/speech-based symptom prediction using ML, but only in terms of treatment outcomes. This is fundamentally not achievable with typical ad hoc observational datasets used in ML studies of the kind that the discipline has so far examined. Diagnostic trials are thus, very likely, the end goal for this line of digital diagnostic assistance technology, if it is to be scientifically reliable for real-world clinical deployment.

## Abbreviations

CV: cross-validation
ML: machine learning
